# Comparison of the Uroflowmetry Parameter Results Between Transgender Males Undergoing Gender-Affirming Hormone Therapy and Age-Matched Cisgender Females: Preliminary Data

**DOI:** 10.1089/trgh.2019.0025

**Published:** 2019-07-01

**Authors:** Kazuna Matsuo, Koji Ichihara, Momokazu Gotoh, Naoya Masumori

**Affiliations:** ^1^ Department of Urology, School of Medicine, Sapporo Medical University, Sapporo, Japan.; ^2^ Department of Urology, Graduate School of Medicine, Nagoya University, Nagoya, Japan.

**Keywords:** androgen, gender-affirming hormone therapy, gender dysphoria, gender incongruence, lower urinary tract function

## Abstract

**Purpose:** Gender-affirming hormone therapy (GHT) using testosterone is administered to transgender males. Although various effects caused by hormonal therapy have been reported, those on lower urinary tract function have remained unclear. The present study compared the uroflowmetry (UFM) parameter results between transgender males and age-matched cisgender females.

**Methods:** A total of 26 transgender males who received GHT for longer than 1 year and the same number of age-matched cisgender females were enrolled. The UFM parameter results and postvoid residual urine volume (PVR) were compared between groups.

**Results:** The median age at enrollment was 31.5 years, and the median duration of hormonal therapy was 2.7 years. There was no significant difference in the maximum flow rate or average flow rate between groups, whereas the mean voided volume (VV) (370±168 vs. 252±73 mL, *p*<0.001) and PVR (57.3±39.5 vs. 19.4±30.5 mL, *p*<0.001) were significantly greater in the transgender male group than those in the cisgender female group.

**Conclusion:** The VV on UFM and postvoided residual urine volume in the transgender males who received GHT for >1 year were significantly increased compared with age-matched cisgender females.

## Introduction

One of the medical interventions for gender-dysphoric/gender-incongruence persons is hormonal therapy. Testosterone enanthate, which is an androgen parenteral preparation, is administered for transgender males as gender-affirming hormone therapy (GHT).^1,2^ Various effects owing to the androgen administration in this population have been reported.^3–7^

Another medical intervention is gender-affirming surgeries. Genital reconstruction as gender-affirming surgery for transgender males is generally composed of hysterectomy and oophorectomy followed by metoidioplasty or phalloplasty. The procedure of metoidioplasty includes urethral elongation using a labia minora flap and anterior vaginal wall flap.^8^ The voiding condition should be different before and after the surgery. The maximum urinary flow rate is decreased after the surgery because of increased urethral resistance due to the urethral elongation.^9^ Postoperative complications such as urethral fistula, urethral stricture, and acute urinary retention may exacerbate the lower urinary tract function or symptoms (LUTS).^10^

For these reasons, it is important to evaluate the lower urinary tract function in transgender persons before genital reconstruction to manage their postoperative condition.^11^ However, to date, the reported information concerning this point is scarce.

Polycystic ovary syndrome (PCOS) is a common endocrine disorder associated with anovulation, elevated circulating androgens, hirsutism, obesity, and insulin resistance.^12^ The etiology of PCOS is still not completely understood, although anovulation and hirsutism are considered as phenotypes of hyperandrogenism. The prevalence of PCOS is higher in transgender males than in the cisgender females.^13^ According to a report which evaluated the relationship between LUTS and serum testosterone levels in cisgender PCOS females, the severity of some LUTS was positively correlated with the serum testosterone levels.^14^ In contrast, there are no data about the association between LUTS and PCOS in transgender males. Moreover, there is no defined information on whether high testosterone exposure in the body affects lower urinary tract function. Although testosterone may play a role in lower urinary tract function and dysfunction across genders and age groups, the overall conclusion is not yet settled.^15^

For these reasons, we conducted a preliminary study. We retrospectively investigated the uroflowmetry (UFM) parameters and postvoid residual urine volume (PVR), which are indexes for lower urinary tract function in transgender males who have received androgen treatment. In addition, we obtained similar parameters from age-matched cisgender females. Then, the mean value of these parameters was compared between groups.

## Methods

A total of 26 transgender males who received GHT and underwent UFM were included. Before transgender persons commence GHT in our hospital, a medical condition check, including blood cell counts and biochemical examination, hormonal tests, and cancer screening, is usually required. In contrast, it is not always necessary to monitor chronological change in each hormonal level after GHT, because the medical fee associated with hormonal therapy for gender-dysphoric/gender-incongruence persons in Japan is not covered by insurance.

Of the 26 subjects, 16 were newly started on GHT in our hospital; five subjects suffered from PCOS defined by the Rotterdam criteria.^12^ In contrast, we could not determine the existence of PCOS in the remaining 10 subjects who started GHT at other hospitals. For GHT, testosterone enanthate intramuscular injection was used. In principle, 250 mg every 2 weeks was selected as the standard dose. If they had an elevated red blood cell count of over 18 g/dL during the GHT administration, the dose and injection interval were modified to avoid erythrocytosis.

Then, we also recruited cisgender females as the control group for comparison with those in the transgender male group. This cohort enrolled 26 healthy volunteers without comorbidity and LUTS: they were matched 1:1 for age, but a difference within 2 years of age was accepted for smooth data collection. All study participants gave written informed consent before study inclusion, and the protocol of this study was approved by the ethics committee of our institution (NO262-48).

First, we retrospectively investigated the following data on transgender males from their medical charts: age at the start of GHT and UFM examination, duration of GHT, the results of UFM and PVR, and subjective symptoms associated with their urination. In addition, the UFM and PVR data in cisgender females were collected. UFM was performed in consideration of privacy in the sitting position in all study participants. Then, we compared these results between the transgender male group and cisgender female group as the primary outcome. Next, we evaluated the relationships between the UFM parameters and the details of GHT. Finally, we evaluated whether the presence of PCOS in transgender males affected the UFM parameter results.

The unpaired *t*-test was used for comparison of the mean values in the statistical analysis in this study. A *p*-value of <0.05 was considered statistically significant.

## Results

### Characteristics of transgender males

All transgender males received GHT for longer than 1 year (Table 1). The median (range) age at the start of GHT was 27 years (18–37). The median age at the time of the UFM examination and median duration of GHT were 30.5 years (22–41) and 2.7 years (1.4–11.7), respectively. The mean baseline testosterone level before GHT administration in the five PCOS patients was 0.71±0.23 ng/mL. This was significantly higher than that in the 11 patients without PCOS (0.44±0.13 ng/mL, *p*=0.027). None of the patients complained of LUTS when they underwent UFM.

**Table 1.** Characteristics of 26 Transgender Males

Parameter	*n*=26
Age at the start of GHT (years)	27.1±4.9, 27 (17–38; years)
Dose of testosterone enanthate (mg)	125 mg; *n*=2, 250 mg; *n*=24
Interval of testosterone injection (every week)	2 (2–4; every week)
Duration of hormonal therapy (years)	4.3±3.1, 2.7 (1.4–11.7; years)
Age at UFM (years)	31.5±5.1, 30.5 (22–41; years)
Diagnosed with PCOS at the start of GHT	Yes; *n*=5, No; *n*=11, Unclear; *n*=10

Data are shown as mean±SD and median (range).

GHT, gender-affirming hormone therapy; PCOS, polycystic ovary syndrome; SD, standard deviation; UFM, uroflowmetry.

### Comparison of the results for UFM and PVR between transgender males and controls

The UFM and PVR results are shown in Table 2. In the transgender males, the mean±standard deviation voided volume (VV) was 370±168 mL. The maximum flow rate (Qmax) was 24.9±9.4 mL/s, and the average flow rate (Qave) was 12.9±4.2 mL/s. The mean and median (range) of PVR were 57.3±39.5 mL and 47.5 mL (0–164), respectively. In comparison with the cisgender females, there was no significant difference in Qmax or Qave. In contrast, the VV and PVR in the transgender males were significantly greater than those in the cisgender females (both *p*<0.001). To confirm whether the simple increased bladder capacity (VV + PVR) affected these results, we calculated %PVR and compared the values between groups (%PVR=100×PVR/bladder capacity). We found that the %PVR in the transgender male group was significantly greater than that in the cisgender group (14.3% vs. 6.2%, *p*=0.003).

**Table 2.** Results of Uroflowmetry and Postvoid Residual Urine Measurement

Parameter	Transgender male (*n*=26)	Control (*n*=26)	p^*^
Age (years)	32±5.2, 30.5 (22–41)	31±5.3, 30.5 (24–42)	0.843
Voided volume (mL)	370±168, 363 (91–836)	252±73, 232 (152–395)	<0.001
Maximum flow rate (mL/s)	24.9±9.4, 24.5 (9.4–50.2)	30.3±10.6, 28.3 (10.9–49.1)	0.084
Average flow rate (mL/s)	12.9±4.2, 13.0 (5.4–20.4)	15.4±5.1, 14.4 (6–24.9)	0.085
PVR (mL)	57.3±39.5, 47.5 (0–164)	19.4±30.5, 0 (0–89)	<0.001
%PVR^**^ (%)	14.3±9.4, 13.2 (0–40.8)	6.2±9.7, 0 (0–31.8)	0.003

Data are shown as mean±SD, median (range).

^*^ Unpaired *t*-test.

^**^ %PVR=100×PVR/(voided volume + PVR).

PVR, postvoid residual urine volume.

### Effects of GHT on UFM and PVR in transgender males

**FIG. 1.**
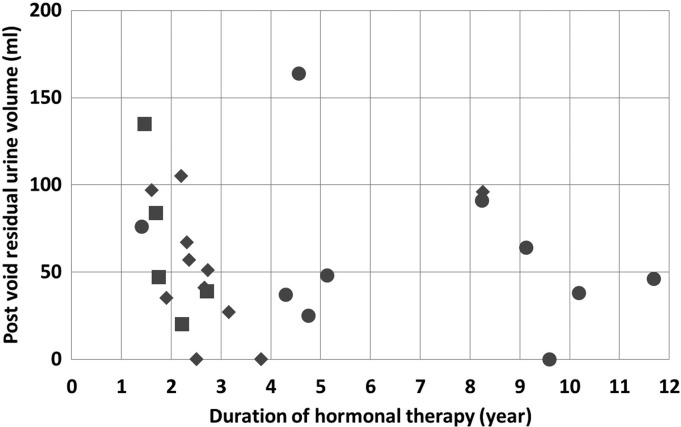
A plot of the results of the GHT duration and the postvoid residual urine volume in each patient. The squares, diamonds, and circles indicate the patients who were diagnosed with PCOS before GHT, diagnosed without PCOS before GHT, and without a diagnosis of PCOS before GHT, respectively. Dividing the patients into groups according to the duration of the GHT, the number of the patients in each group was as follows: ≤2 years, 6; >2 years, 20; ≤3 years, 14; and >3 years, 12. GHT, gender-affirming hormone therapy; PCOS, polycystic ovary syndrome.

The effect of GHT duration on PVR was evaluated (Fig. 1). When the subjects were divided into two groups according to the duration of hormonal therapy (≤2 or >2 years) and (≤3 or >3 years), there was no significant difference in PVR between the groups (*p*=0.135 and *p*=0.623, respectively). Even in the six transgender males who received androgen treatment for <2 years, the PVR was 79±32.8 mL. Next, we evaluated whether the presence of PCOS, which is, in other words, the difference in the baseline endogenous testosterone level, in transgender males, affected the UFM parameter results. The mean durations of GHT in the five PCOS and the 11 non-PCOS subjects were 2.0 and 3.1 years, respectively (*p*=0.092). No significant difference was observed between the two groups for the total dose of testosterone (*p*=0.340). The VV, Qmax, and Qave in the PCOS and non-PCOS subjects were 435±129 mL and 305±138 mL, 27.5±6.0 mL/s and 22.9±9.0 mL/s, and 13.8±2.3 mL/s and 12.8±3.8 mL/s, respectively. There were no significant differences between the groups for these parameters (VV, *p*=0.134; Qmax, *p*=0.290; and Qave, *p*=0.585), and there was also no significant difference in PVR (PCOS, 65.0±40.7 mL; non-PCOS, 52.4±35.0 mL, *p*=0.604).

## Discussion

Simple hysterectomy and oophorectomy followed by metoidioplasty are one standard genital reconstruction technique for transgender males.^1–3,8,10^ Since their postoperative voiding condition is changed, its chronological evaluation is extremely important.^9,11^ However, there are no data on whether GHT using testosterone itself affects the lower urinary tract function in transgender males. Although this is a retrospective/preliminary study, it is the first study to evaluate the lower urinary tract function of transgender males who received GHT for >1 year.

With respect to lower urinary tract function in healthy adult cisgender females, Barapatre et al.^16^ reported that the mean VV and PVR in 308 females with a mean age of 33 years were 289.79±166.52 mL and 2.92±3.69 mL, respectively. Similarly, Karsenty et al.^17^ demonstrated that the mean PVR in 30 cisgender females (mean age was 39 years) was 29 mL in the seated position and 38 mL in the standing position. In line with these previous data, our results in the cisgender females showed that the mean VV and PVR were 252±73 mL and 19.4±30.5 mL, respectively. Thus, PVR in females was small, usually <50 mL. In contrast, the normal range of VV is difficult to explain. At the least, we thought that VV data in both groups of the present study were not abnormal. However, there was a significant difference in VV between the groups. Although there have been no data about the bladder capacity and residual urine volume in human females who received androgen treatment, one animal experiment using mice showed a result similar to our findings.^18^ In that study, testosterone-treated ovary-intact mice had larger PVR and greater bladder/kidney weights than ovary-intact females. The authors concluded that testosterone might induce morphological modifications in the urinary tract. However, histological examination showed no significant difference in the bladder muscle thickness between with and without testosterone treatment. In general, sex difference in the lower urinary tract function exists across species. For example, VV and bladder weight per body in male mice are larger than those in female mice.^18^ Male mice experienced more micturition than female mice, thus suggesting a sex difference in mouse micturition pattern.^19^ In humans, the expression of muscarinic receptors, which are the key factors for sensation and detrusor function in the bladder, differs with age and gender.^20^ In our study, we clarified that the transgender males who received GHT for longer than 1 year had increased VV and PVR compared to the cisgender females of comparable age. Therefore, high androgen exposure may modify the voiding pattern from female to male type in transgender males.

PCOS is a condition characterized by high levels of serum testosterone. Baba et al.^13^ reported that the prevalence of PCOS was higher in the transgender males than in the cisgender females. Sahinkanat et al.^14^ examined the relationship between LUTS and serum testosterone levels in 140 cisgender PCOS females. They found that the severity of nocturia, dyspareunia, pelvic pain, and urinary urgency was positively correlated with the serum testosterone levels. However, no significant correlation was observed between the PVR and serum testosterone level. Serum testosterone levels may be much higher in the transgender males with PCOS after receiving GHT than those in the GHT-naive PCOS patients or the transgender males without PCOS. Thus, we hypothesized that the transgender males with PCOS might have a more prominent imbalance in micturition than without PCOS. As a result, there was no significant difference in the urinary flow rate and PVR between with and without PCOS. However, we evaluated too few subjects. Further evaluation using a large number of subjects should be conducted to clarify the effects on the lower urinary tract function in transgender males with PCOS.

In the present study, the duration of GHT did not affect the PVR results. Impairment of the lower urinary tract function by GHT might appear within a short period of time after starting androgen treatment. However, we could not confirm the early effects of GHT on lower urinary tract function because all subjects in this cohort received GHT for longer than 1 year.

Finally, the limitations should be addressed. First, the number of subjects in the present examination was small, and the data collection for UFM and PVR in the transgender males was performed retrospectively. Second, we evaluated VV on UFM and PVR as lower urinary tract functions only once. We did not assess LUTS using a validated questionnaire. Moreover, there are differences between subjects and the control group besides having or not having GHT. There are other possible differences that may affect voiding conditions such as barriers to bathroom use for transgender people and sociocultural/socioeconomic factors. Therefore, GHT may be a primary possible factor that moderates voiding parameters. Finally, there were no baseline data on UFM and PVR before GHT. The chronological changes in serum testosterone, estrogen, and other hormones were not measured in all transgender males after GHT.

Considering these limitations, a prospective study must be conducted to evaluate the relationship between the sequential changes in lower urinary tract function and the serum levels of sex hormones in transgender males who received GHT. However, our study is the first and only one to evaluate the voiding condition and PVR in transgender males receiving androgen therapy. If one of the final stages of the treatment for transgender persons is gender-affirming surgery, including genital reconstruction, it will be necessary to investigate the effect of GHT on lower urinary tract function. In conclusion, the VV and PVR in transgender males who received GHT using testosterone for >1 year are increased compared with those in age-matched cisgender females.

## Authors' Contributions

Category 1: Conception and design (K.M., K.I., and N.M.), acquisition of data (K.M. and K.I.), and analysis and interpretation of data (K.M. and K.I.). Category 2: Drafting the article (K.M. and K.I.) and revising it for intellectual content (M.G. and N.M.). Category 3: Final approval of the completed article (K.I. and N.M.).

## Abbreviations Used

GHT: gender-affirming hormone therapy
LUTS: lower urinary tract symptoms
PCOS: polycystic ovary syndrome
PVR: postvoid residual urine volume
Qave: average flow rate
Qmax: maximum flow rate
SD: standard deviation
UFM: uroflowmetry
VV: voided volume
